# Postpartum depression-associated localized neural dysfunction: a voxel-wise meta-analysis of amplitude and synchronization alterations in resting-state fMRI

**DOI:** 10.3389/fpsyt.2025.1660550

**Published:** 2025-10-08

**Authors:** Jingjing Qiu, Hongjin Wang, Yu Zhao, Shen Lin, Shangjie Chen

**Affiliations:** ^1^ Department of Rehabilitation Medicine, The People’s Hospital of Baoan Shenzhen, Shenzhen, China; ^2^ Department of Rehabilitation Medicine, The Second Affiliated Hospital of Shenzhen University, Shenzhen, China; ^3^ Department of Rehabilitation Medicine, Zhongxian People’s Hospital of Chongqing, Chongqing, China; ^4^ Postgraduate College, Guangzhou University of Chinese Medicine, Guangzhou, China; ^5^ Department of Traditional Chinese Medicine, Fujian Maternity and Child Health Hospital, Fuzhou, China

**Keywords:** postpartum depression, voxel-wise meta-analysis, resting-state fMRI, localized neural dysfunction, intrinsic neural dynamics

## Abstract

**Background:**

Resting-state fMRI studies in postpartum depression (PPD) have reported voxel-wise alterations in measures of neural amplitude and synchronization, yet scarce meta-analysis has quantitatively synthesized these findings. We performed a coordinate-based meta-analysis to identify convergent amplitude and synchronization dysfunction in PPD.

**Methods:**

We conducted a comprehensive search for whole-brain voxel-wise resting-state fMRI studies comparing PPD patients with healthy postpartum controls that reported local amplitude or synchronization metrics. Peak coordinates were analyzed using the Anisotropic effect size-signed differential mapping to delineate whole-brain functional alterations.

**Results:**

Ten studies (288 PPD patients, 279 controls) contributed 62 peak foci. Our analysis revealed that PPD patients exhibited increased activity in the left fusiform gyrus (FFG.L), left middle occipital gyrus (MOG.L), while showing decreased activity in the left anterior cingulate gyrus (ACG.L), the right superior temporal gyrus (STG.R), the right insula (INS.R) and the right precentral gyrus (PreCG.R) compared to healthy subjects. Jackknife sensitivity analysis indicated minimal impact on the overall results when eliminating any single study. Meta-regression analysis revealed a correlation between MOG.L functional activity and Edinburgh postnatal depression scale scores.

**Conclusion:**

Abnormally elevated functional activity in the FFG.L, MOG.L, along with reduced activity in the ACG.L, STG.R, INS.R and PreCG.R, may serve as potential biomarkers for PPD. Additionally, abnormal functional activity in the visual cortex, and the prefrontal cortex-limbic system may be associated with PPD.

## 1 Introduction

Postpartum depression (PPD), marked by the presence of depressive symptoms 4–6 weeks after childbirth, is a severe psychiatric complication in the postnatal period (1). PPD is characterized by enduring feelings of sadness, cognitive impairment, significant memory and concentration issues, a noticeable decline in motivation, a lack of interest in activities, reluctance to engage in communication, and physical symptoms such as tightness in the chest, loss of appetite, frequent regurgitation, nausea, and disruptions in sleep patterns (2). The Edinburgh Postnatal Depression Scale(EPDS) and Hamilton Depression Scale(HAMD) were commonly used for measurements of PPD (3–5).

According to Wang et al. (6), the global prevalence of PPD is approximately 17%. In a cohort study by Tebeka (7), the reported incidence of early PPD at eight weeks was 8.3%, while the late incidence at one year was 12.9%. Furthermore, households’ medical costs are 22% higher for women with PPD compared to unaffected postpartum women (8). PPD not only reduces the quality of life but also increases the economic burden on individuals (9). Overall, PPD is a significant global public health issue that requires early identification and treatment to improve maternal and child health outcomes.

However, the pathogenesis of PPD remains unclear. A better understanding of the pathogenesis of PPD can lead to more effective treatment strategies. Three standard metrics, namely, the amplitude of low-frequency fluctuations (ALFF), fractional ALFF (fALFF), and regional homogeneity (ReHo), are commonly used in resting-state fMRI studies (10). These metrics provide complementary information about spontaneous brain activities in different regions and reflect their interaction, contributing to our understanding of brain activity (11, 12). The utilization of ALFF, fALFF and ReHo enables the identification of functional abnormalities associated with PPD, which provides valuable insights into altered spontaneous brain activity.

In comparison to healthy participants, individuals with PPD exhibited notable alterations in brain activity patterns. Specifically, decreased amplitude of ALFF was observed in the ACC.R, alongside increased ALFF in the left calcarine. Differences were also noted in degree centrality (DC) with decreased values in the right middle cingulate cortex(MCC.R) and increased values in the left fusiform gyrus(FFG.L). Additionally, decreased ReHo was found in the ACC.R, while increased ReHo was observed in the middle occipital gyrus(MOG.L). The overlap analysis of these functional measures revealed clusters of significance in the left inferior occipital gyrus (IOG.L) and the ACC.R region. Another PPD cohort study demonstrated increased ReHo in the left precuneus(PCUN.L) and right hippocampus(HIP.R), whereas reduced ReHo was observed in the dlPFC and right insula(INS.R). In the comparison of whole-brain ALFF between the PPD and the control group, ALFF in the precentral gyrus(PreCG) regions of the PPD group significantly decreased, while ALFF in the INS and superior temporal gyrus(STG) regions significantly increased, with no statistical difference in the remaining brain regions.

However, relevant resting-state fMRI studies have reported inconsistent results. Previous research (13) found that the ALFF values were decreased in the bilateral dlPFC and insula lobes in PPD patients. On the contrary, Che kaili and co-workers (14) showed that the ALFF values were higher in the left dlPFC of PPD patients compared to healthy controls. Schnakenberg and colleagues (15) demonstrated no differences in voxel-wise or spontaneous functional brain activity between PPD and healthy women.

FMRI studies of PPD vary widely in outcomes, influenced by factors such as sample size, study design, imaging protocols, and participant characteristics. These variabilities challenge the interpretation of fMRI findings in PPD, highlighting a need for a comprehensive synthesis of available data. To address the potential sources of variability, we conducted a meta-analysis of existing clinical fMRI studies, aiming to delineate whole-brain voxel-level changes in functional brain activity among PPD patients. This analysis included peer-reviewed fMRI studies that encompass both whole-brain scanning and examinations of resting-state brain activities. By aggregating and scrutinizing these studies, our research seeks to provide a more reliable and nuanced understanding of the neural underpinnings of PPD, offering insights that could potentially guide more effective diagnostic and therapeutic strategies. In addition to the meta-analytic identification of convergent neural alterations in PDD, we further employed hierarchical clustering analysis (HCA) to characterize the spatial distribution of reported foci. This complementary step allows us to examine not only the common abnormalities across patients but also potential variations in relation to postpartum course.

## 2 Methods

### 2.1 Literature search and selection criteria

The meta-analysis adhered to the Meta-analyses Of Observational Studies in Epidemiology (MOOSE) Checklist (**Supplementary Table 1**), and was registered in PROSPERO with the registration number CRD42022345362. A comprehensive search was conducted in PubMed, Embase, and Web of Science datasets. The search items for postpartum depression included (“Postpartum Depressive” OR “PPD” OR “Postnatal Depression” OR “Depression, Postnatal” OR “Postpartum Depression” OR “Depression, Postpartum” OR “Post Partum Depression” OR “Postpartum Depression” OR “Postnatal Depression” OR “Depression, Postnatal” OR “Post Natal Depression”). The search terms about neuroimaging were (“fMRI” OR “Functional magnetic resonance imaging” OR “functional MRI” OR “BOLD” OR “Blood oxygen level-dependent” OR “regional homogeneity” OR “ReHo” OR “amplitude of low-frequency fluctuations” OR “ALFF” OR “fractional amplitude of low-frequency fluctuations” OR “fALFF” OR “brain activity” OR “brain function” OR “resting state” OR “rs”) (**Supplementary Table 2**). Similar search terms were translated into Chinese for the identification of studies in Chinese databases including CNKI, WanFang Data, and China Science and Technology Journal Database (VIP).

Two independent researchers, JJQ and HJW, conducted the literature search in all six databases from the establishment of the database until December 25, 2024. The inclusion criteria for identified studies were as follows: (1) fMRI studies that compared PPD patients with healthy controls, (2) the use of resting-state fMRI scanning methods, (3) reporting of peak coordinates in the Talairach or Montreal Neurological Institute (MNI) standard space coordinate system, (4) whole-brain-based fMRI analysis, and (5) articles written in English or Chinese.

Studies were excluded based on the following criteria: (1) duplicate publications, (2) non-case-control studies or non-clinical studies, (3) conference papers or dissertations, (4) case reports/protocol, reviews, or meta-analyses; (5) studies that utilized only the region of interest (ROI) or voxel-mirrored homotopic connectivity (VMHC) methods, (6) studies that were not fMRI or not related to PPD, and (7) studies with a sample size of less than 10. In cases where studies had overlapping samples, only the study with the largest sample size was selected. Additional studies were identified through the reference lists of the identified literature.

### 2.2 Selection of studies

JJQ and HJW independently conducted the searches in each database, and the search results were imported into EndNote X9 reference management software for efficient organization and subsequent screening. After removing any duplicate publications, JJQ and HJW independently screened the remaining literature based on the information provided in the titles and abstracts. In cases where the abstracts did not provide sufficient information to determine inclusion or exclusion, the researchers retrieved and reviewed the full text of the articles according to the predefined inclusion and exclusion criteria.

Throughout the screening process, JJQ and HJW engaged in regular discussions to exchange their perspectives and address any disagreements or uncertainties. If needed, the team’s third researcher (SL) participated in the discussion to provide further input and help resolve any conflicts. This systematic screening process ensured a thorough and unbiased selection of studies for the meta-analysis while incorporating multiple researchers’ perspectives and promoting consensus on study inclusion.

### 2.3 Quality assessment and data extraction

The Cochrane Risk of Bias Assessment Tool Newcastle-Ottawa Scale (NOS) was utilized to evaluate the risk of bias in the studies included in the meta-analysis (16). Two authors (JJQ and HJW) independently extracted relevant data from the selected studies. A total of 10 studies were included in the analyses. The extracted data included general information (first author, group, participants, title, journal, publication year), study design (sample size, analysis methods, depression scale score, postpartum time, multiple comparison correction, statistical threshold, and software package). The *x*, *y*, and *z* coordinates of statistically significant results observed in the PPD group compared to the healthy control subjects (HCs), reported in either Talairach or MNI space.

### 2.4 Voxel-wise meta-analysis

The meta-analysis was performed using Anisotropic effect size-signed differential mapping (AES-SDM) version 6.22 (http://www.sdmproject.com/software). Initially, peak coordinates and threshold values of the contrast between PPD patients and healthy controls were extracted from the included studies. In cases where the original papers reported z-scores instead of t-statistics, the z-scores were converted to t-statistics using an online converter (https://www.sdmproject.com/utilities/?show=Statistics). All Talairach coordinates were converted to MNI152 space using GingerALE (17).

A voxel-level meta-analysis of the whole brain was conducted using functional MRI or PET as the preprocessing modality and GM as the correlation template. The analysis involved 50 randomizations, and an anisotropy value of 1 was set (18). Firstly, the raw data was transformed into standardized effect sizes using Cohen’s d, calculated based on the mean and standard deviation of the control group. Subsequently, these effect sizes were smoothed using a Gaussian kernel with a full-width at half-maximum (FWHM) value of 20 mm (19). Finally, the effect sizes were converted into z-scores, utilizing the mean and standard deviation of the entire dataset.

To account for sample size and variations between studies, a random-effects model was utilized. Effect sizes were then calculated for each study based on the extracted data. The random-effects model considers the heterogeneity between studies by assigning weights to each study when determining the synthetic effect size. Studies with larger sample sizes are given higher weights, while studies with less heterogeneity contribute more to the overall synthetic effect size. The significance threshold was set at p<0.005, with a minimum peak height of 1 and a minimum cluster size of 10 voxels (19, 20). Furthermore, a meta-regression analysis was performed, incorporating maternal age and EPDS as regression factors.

### 2.5 Sensitivity analysis, heterogeneity examination, and publication bias assessment

We have finished the Jackknife sensitivity analyses and publication bias assessment on the results of the resting-states. Jackknife sensitivity analysis was conducted by systematically removing one study at a time from the entire dataset and recalculating the model or estimate. This process allowed us to assess the sensitivity of the results to the inclusion of each individual study (21). Statistical heterogeneity refers to the variability in intervention effects observed across the included studies, resulting from differences in research types, participants, interventions, outcomes, designs, and potential biases. To evaluate heterogeneity, we employed the random-effects model and utilized Q statistics (22). A higher Q value indicates greater variation and heterogeneity between studies. When employing the random effects model, the weights of each study are adjusted to account for this heterogeneity, leading to more accurate results. Effect sizes, standard errors, standardized effect sizes, and overall effect size estimates were calculated for each individual study. To calculate the weight values for each study, the standard errors and tau-squared values were combined. These weights, along with the effect sizes, were then used to calculate the overall study heterogeneity tau-squared value, which indicates the degree of heterogeneity in the effect sizes across the entire study-in other words, the level of variation between the results of different studies. For each study, a p-value was calculated based on the z-score and degrees of freedom. Following standardization, AES-SDM analysis was performed to generate spatial distribution maps and cluster analysis results. The significance threshold was set at *p*<0.005, with a minimum peak height of *Z* 1 and a minimum cluster size of ten voxels. Furthermore, we constructed a mask using the coordinates obtained from the primary analysis results. The mask was used to extract the effect sizes specifically associated with the identified brain regions. To assess publication bias, we employed a funnel plot and conducted the Egger test, and *p*<0.05 was considered statistically significant.

### 2.6 Hierarchy clustering analysis

Hierarchical clustering analysis (HCA), an unsupervised machine learning algorithms, was conducted using the scipy.cluster.hierarchy.linkage in Python to explore and understand the patterns within brain coordinate datasets (23). HCA proves valuable in identifying natural groupings within datasets, highlighting brain regions that share similar functional changes across the studies included. Euclidean distance (ED) is commonly used as a measure to quantify the distance between two foci, and is also frequently employed in HCA to assess similarity. Among the 10 included studies, there were a total of 62 focal coordinates. We calculated the ED between all pairs of foci and merged the closest clusters based on the distance. A tree-like structure (dendrogram) was constructed to visually display the relationships among different clusters. The threshold was established as 70% of the maximum distance that separates the clusters (24). In addition, based on whether the postpartum period is full 1 year or not, we categorized the included studies into two major types. We used a correlation score (*L*) to evaluate the relationship between the coordinates from different postpartum periods and the clusters of HCA results. This score was calculated using the following equation:


$$L_{ij}=\;r_{ij}/R_{i}$$

$$r_{ij}=\;m_{ij}/M_{j}$$

$$R_{j}=\;n_{j}/N$$

*M_ij_* represents the coordinate counts of the *i* ^th^ postpartum periods-triggered pattern in the *j* ^th^ cluster, *M_j_* is the coordinate count of the *j* ^th^ cluster, and *r_ij_* denotes the proportion of coordinates for the *i* ^th^ postpartum periods-triggered pattern in the *j* ^th^ cluster. *n_i_* is the coordinate counts of the *i* ^th^ postpartum periods-triggered pattern, *N* is the total coordinate counts of the postpartum periods dataset, and *R_i_* indicates the proportion of coordinate for the *i* ^th^ pattern in the total coordinate count (25). A larger *L* score indicates a stronger association between a specific cluster and a particular postpartum periods-triggered pattern (26).

## 3 Results

### 3.1 Participant demographics of the included studies

Ten studies were included in this meta-analyses, and the NOS tool was used to evaluate the quality of these studies. Based on the scoring criteria, all the included studies were considered moderate to high quality. This indicates that the included studies were of good quality in terms of their study design, sample selection, and outcome assessment, which provides reliable primary data for the subsequent analysis (**Table 1**). We employed an additional quality assessment step using a 20-point checklist (**Supplementary Table 3**), previously used in fMRI meta-analyses (**Table 2**) (35–37).

**Table 1:** Literature quality assessment results.

Study	Select			Comparability		Exposure			Scores
	Adequate definition of cases	Representat- ivenness of the cases	Selection of controls	Definition of controls	Control for important factor	Ascertainment of exposure	Same method of ascertainment for cases and controls	No response rate	
Xu et al. (27)	**✵**	**✵**	**✵**	**✵**	**✵**	**✵**	**✵**	–	7
Li et al. (28)	**✵**	**✵**	**✵**	**✵**	**✵✵**	**✵**	**✵**	**✵**	9
Li Bo (29)	**✵**	**✵**	**✵**	**✵**	**✵✵**	**✵**	**✵**	–	8
Dong et al. (30)	–	**✵**	**✵**	**✵**	**✵✵**	**✵**	**✵**	**✵**	8
Zhang et al. (31)	**✵**	**✵**	–	**✵**	**✵✵**	**✵**	**✵**	**✵**	8
Xianv et al. (32)	**✵**	**✵**	–	**✵**	**✵✵**	**✵**	**✵**	**✵**	8
Che et al. (14)	**✵**	**✵**	**✵**	**✵**	**✵✵**	**✵**	**✵**	**✵**	9
Xiao-juan et al. (33)	**✵**	**✵**	–	**✵**	**✵**	**✵**	**✵**	–	6
Dong et al. (13)	**✵**	**✵**	–	**✵**	**✵**	**✵**	**✵**	**✵**	7
Cheng et al. (34)	**✵**	**✵**	–	**✵**	**✵✵**	**✵**	**✵**	**✵**	8

The Newcastle-Ottawa Scale (NOS) tool is used to evaluate the quality of included studies and the scores ranging from 0 to 9 represent increasing quality.**✵**” indicates that 1 point is awarded for the corresponding item, and “**✵**” indicates that 2 points are awarded.

**Table 2:** Demographic and clinical characteristics of included studies.

Study	Methods	Software package	Subject (F)		Mean age (SD)		Edinburgh		Postpartum time (days)	Statistical threshold	Quality Scores
			PDD	HC	PDD	HC	PDD	HC			
Xu et al. (27)	ALFF, DC, and ReHo	SPM12	52	24	32.73(3.93)	32.41(4.26)	15.51 (5.24)	4.37 (3.24)	within 1 year after birth	GRF (p < 0.01) and a cluster level of p < 0.05	17
Li et al. (28)	ReHO	SPM12	28	29	29.27 (4.72)	28.56 (4.57)	14.97 (1.66)	0.79 (0.96)	in the fourth week after delivery	p<0.05,FDR corrected,cluster size > 55 voxels	16
Li Bo (29)	ALFF	SPM12	35	34	27.11(3.71)	26.83(3.90)	12.93(2.48)	0.79(0.94)	Within 6 weeks postpartum	p<0.05, GRF corrected	16
Dong et al. (30)	dALFF	DPARSF	24	19	31.05 (2.96)	30.95 (4.39)	17.35 (2.70)	2.67 (0.19)	NA	p<0.05, GRF corrected	16
Zhang et al. (31)	DC	SPM12	29	30	27.24 (3.55)	27.33 (4.10)	15.79 (1.86)	0.50 (0.73)	in the fourth week after delivery	voxel p < 0.001,cluster p<0.05,2-tailed GRF corrected	16
Xianv et al. (32)	fALFF	SPM12	23	28	28.9 (4.4)	30.5 (4.0)	13	2	Within 8 weeks postpartum	AlphaSim, p<0.05	15
Che et al. (14)	fALFF and ReHo	SPM8	16	16	31.16 (2.56)	31.06 (4.42)	16.13 (3.34)	2.61 (0.31)	1 year after childbirth	p< 0.005, FDR corrected	16
Xiao-juan et al. (33)	ReHO	SPM8	10	11	27.58 (4.56)	27.16 (3.68)	NA	NA	within 16 weeks of giving birth	Voxels with a p value<0.01 (uncorrected) and cluster size> 10 voxels	13
Dong et al. (13)	ALFF	SPM	26	26	26.8 (2.2)	27.7 (3.7)	18.3 (5.1)	2.5 (0.1)	Within 6 weeks postpartum	AlphaSim, p<0.05	15
Cheng et al. (34)	dALFF	SPM	45	62	31.11 (3.19)	32.42 (3.92)	16.2 (3.22)	7.06 (4.14)	within 1 year after birth	corrected threshold of p < 0.05 (cluster-forming threshold at voxel-level p < 0.001	15

ReHO, Regional homogeneity; fALFF, fractional amplitude of low-frequency fluctuation; dALFF, dynamic amplitude of low-frequency fluctuation; DC, degree centrality; GRF, Gaussian Random Field; FEW, Family-Wise Error Rate; FDR, False Discovery Rate; PDD, postpartum depression; HC, healthy control.

**Figure 1:**
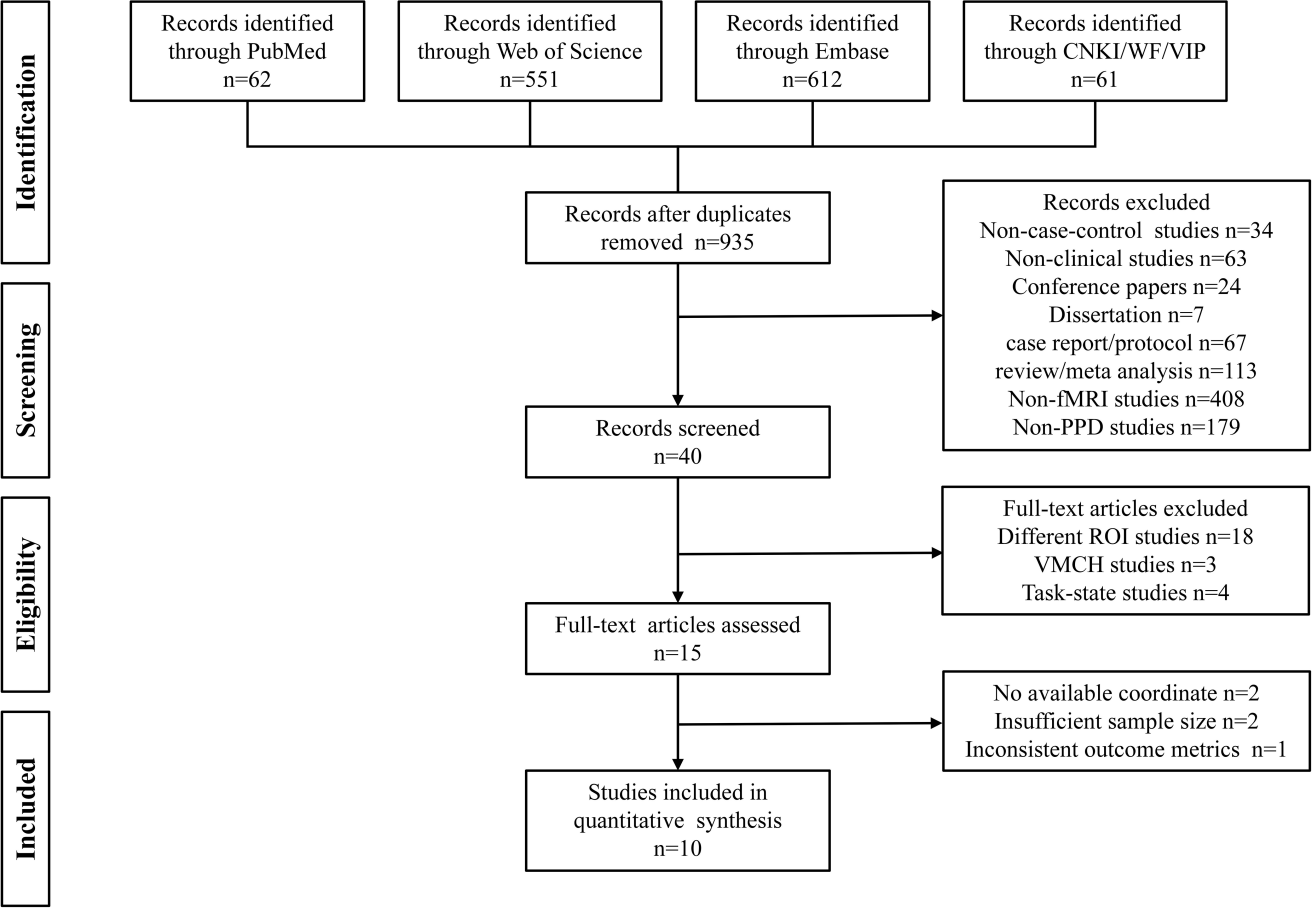
Flowchart illustrating the search results and study selection process.

The 10 studies involved a total of 288 patients with PPD, with a mean age of 29.66 years old. Additionally, there were 279 healthy controls included in the studies, with a mean age of 29.77 years old (**Table 2**, **Figure 1**). Regarding the time period after delivery, seven studies (13, 27–29, 31, 32, 34) examined PPD patients within one year of delivery; two studies (14, 33) assessed PPD patients over one year of postpartum; and one study did not report the specific postpartum time (30). Among the ten resting-state studies, two focused on dALFF (30, 34), two on fALFF (14, 32), four on ReHo (14, 27, 28, 33), three on ALFF (13, 27, 29), and two on DC (27, 31) (**Table 2**).

### 3.2 The analysis results for resting-state studies on PPD and HCs

#### 3.2.1 Differences in the whole brain’s functional activities

**Figure 2:**
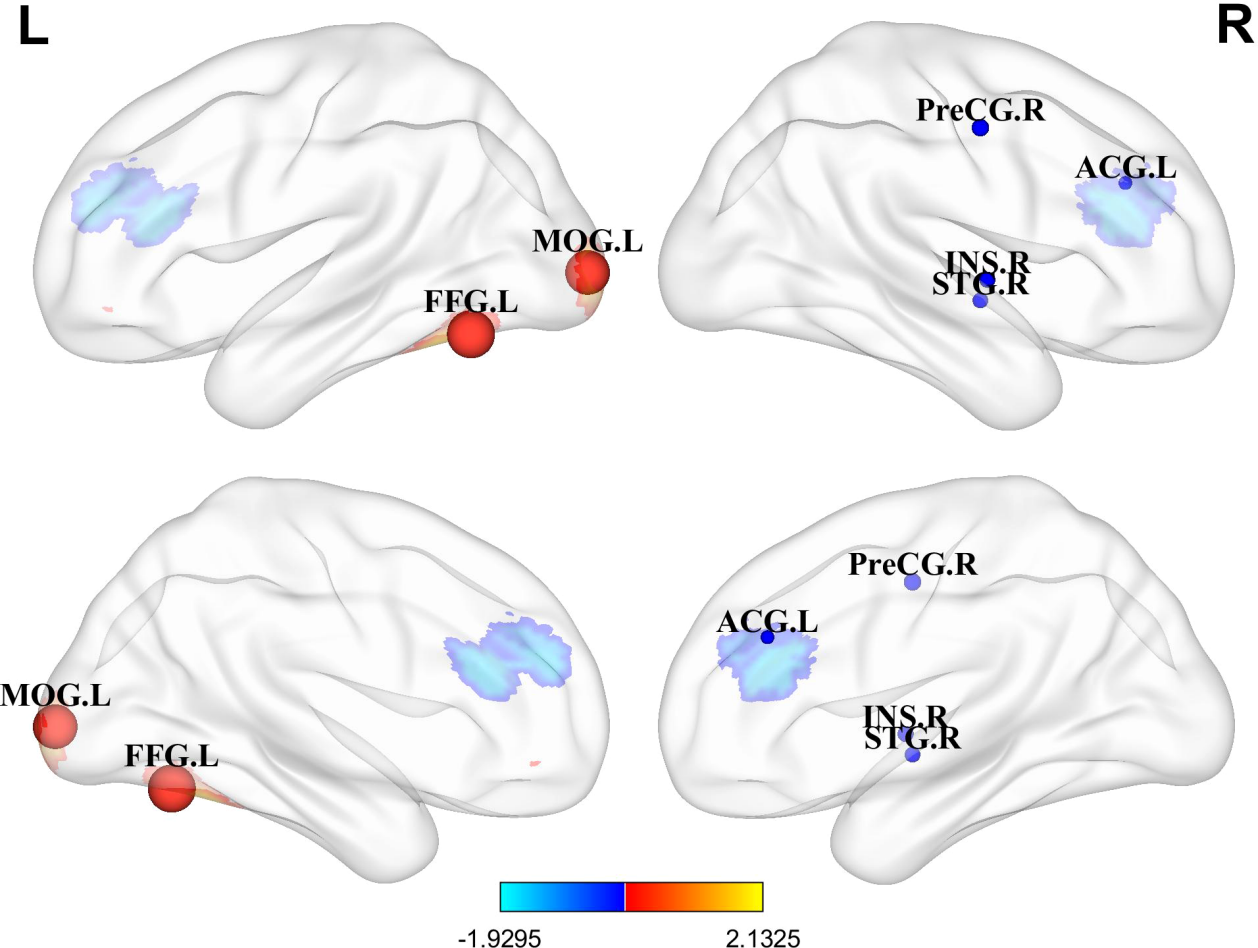
Differences in brain functional activities between postpartum depression (PPD) patients and healthy controls (HCs). Regions displaying altered regional brain activity in PPDs compared to HCs are highlighted. The significance threshold was set at p<0.005, with a minimum peak height of 1 and a minimum cluster size of 10 voxels. Red indicates increased activity, while blue indicates decreased activity. FFG.L, Left fusiform gyrus; MOG.L, Left middle occipital gyrus; ACG.L, Left anterior cingulate; STG.R, Right superior temporal gyrus; PreCG.R, Right precentral gyrus; INS.R, Right insula.

In resting-state studies, PPD patients demonstrated increased neural activity in the FFG.L and the MOG.L. They also exhibited decreased neurofunctional activity in several regions, including the ACG.L, STG.R, PreCG.R and INS.R (**Table 3**, **Figure 2**).

**Table 3:** Regions with abnormal functional brain activities in PPD patients compared with HCs.

Anatomical lable	MNI coordinate	Voxels	SDM-Z	Effect size	P value	Heterogeneity	Egger test (P value)
Left fusiform gyrus	-44,-62,-16	883	1.866	0.17	0.000	No	0.872
Left middle occipital gyrus	-22,-96,2	342	1.511	0.25	0.002	No	0.810
Corpus callosum	-12,32,-12	173	1.872	-0.62	0.000	No	0.553
Left cerebellum, hemispheric lobule VIII	-8,-70,-34	907	-1.614	-1.01	0.000	No	0.340
Left anterior cingulate/paracingulate gyri	2,34,28	513	-1.721	0.48	0.000	No	0.644
Right superior temporal gyrus	44,-8,-6	103	-1.516	-0.60	0.001	No	0.563
Right insula	42,-6,0	103	-1.469	-0.71	0.002	No	0.497
Right precentral gyrus, BA 6	52,-8,44	22	-1.335	-1.89	0.001	No	0.096

BA, Brodmann area; MNI, Montreal Neurological Institute; SDM, seed-based d mapping.

#### 3.2.2 Analyses of sensitivity, heterogeneity, and publication bias

Regarding heterogeneity analysis, the results indicated that there was no significant heterogeneity in the seven regional coordinates of abnormal brain function observed in PPD patients. Furthermore, the Egger test, which assesses publication bias, found no evidence of bias in the analysis results, indicating that the included studies were not disproportionately influenced by publication or reporting biases (**Table 3**, **Supplementary Figure 1**).

The results of Jackknife sensitivity analysis indicated that removing any single article had little effect on the overall results, suggesting that the conclusions drawn from the meta-analysis were not significantly influenced by any specific study. Among the 10 studies, 7 showed elevated activities in the FFG.L, 8 in the MOG.L, indicating increased neural activity in these regions among PPD patients. Additionally, 8 studies demonstrated decreased functional activity in the ACG.L, INS.R and the STG.R. Similarly, 7 studies displayed reduced functional activity in the PreCG.R (**Table 4**). To explore the potential factors contributing to heterogeneity, a meta-regression analysis was conducted using maternal age and EPDS as regression factors. However, the results indicated that both maternal age and depression severity did not significantly influence heterogeneity in the meta-analysis results (**Table 5**).

**Table 4:** Results of Jackknife sensitivity analysis of resting-state studies.

Study	Increased			Decreased			
	Left fusiform gyrus	Left middle occipital gyrus	Corpus callosum	Left anterior cingulate/paracingulate gyri	Right superior temporal gyrus	Right insula	Right precentral gyrus, BA 6
Xu et al. (27)	⨯	⨯	✓	⨯	✓	✓	✓
Li et al. (28)	✓	✓	✓	✓	⨯	⨯	✓
Li Bo (29)	⨯	✓	✓	⨯	✓	✓	✓
Dong et al. (30)	✓	✓	✓	✓	✓	✓	⨯
Zhang et al. (31)	✓	✓	✓	✓	✓	✓	✓
Cheng et al. (34)	✓	✓	✓	✓	✓	✓	⨯
Xianv et al. (32)	✓	⨯	✓	✓	✓	✓	✓
Che et al. (14)	✓	✓	✓	✓	✓	✓	⨯
Dong et al. (13)	⨯	✓	✓	✓	⨯	⨯	✓
Xiao-juan et al. (33)	✓	✓	✓	✓	✓	✓	✓
total	7/10	8/10	10/10	8/10	8/10	8/10	7/10

**Table 5:** Meta-regression analysis of resting state studies.

Index	MNI coordinate	SDM-Z	P	Voxels	Description
Edinburgh scale scores	-30,-96,2	2.103	0.001	335	Left middle occipital gyrus, BA 18
	-52,-68,-14	2.029	0.002	131	Left inferior occipital gyrus, BA 37
	-6,30,16	-2.211	0.002	97	Left median network, cingulum
	8,-62,20	-2.000	0.004	11	Right cuneus cortex, BA 23
	-42,-6,-6	-2.035	0.001	373	Left insula,BA 48
	44,-8,-2	-1.993	0.001	247	Right insula,BA 48
	44,-14,12	-1.964	0.002	247	Right superior temporal gyrus,BA 48
	18,66,22	-2.063	0.009	92	Right superior temporal gyrus,dorsolateral,BA 10
Age	-18,-94,4	2.633	0.000	711	Left optic radiations
	-50,-64,-20	2.424	0.001	217	(undefined), BA 37
	-12,32,-10	1.929	0.004	27	Corpus callosum
	-6,-68,-40	-2.835	0.000	1073	Left cerebellum, hemispheric lobule VIII
	-6,-2,30	-2.137	0.004	25	Left median network, cingulum
	-20,-100,-4	2.630	0.000	716	Left middle occipital gyrus, BA 18

BA, brodmann area; MNI, Montreal Neurological Institute; SDM, seed-based d mapping.

### 3.3 Hierarchy clustering analysis

**Figure 3:**
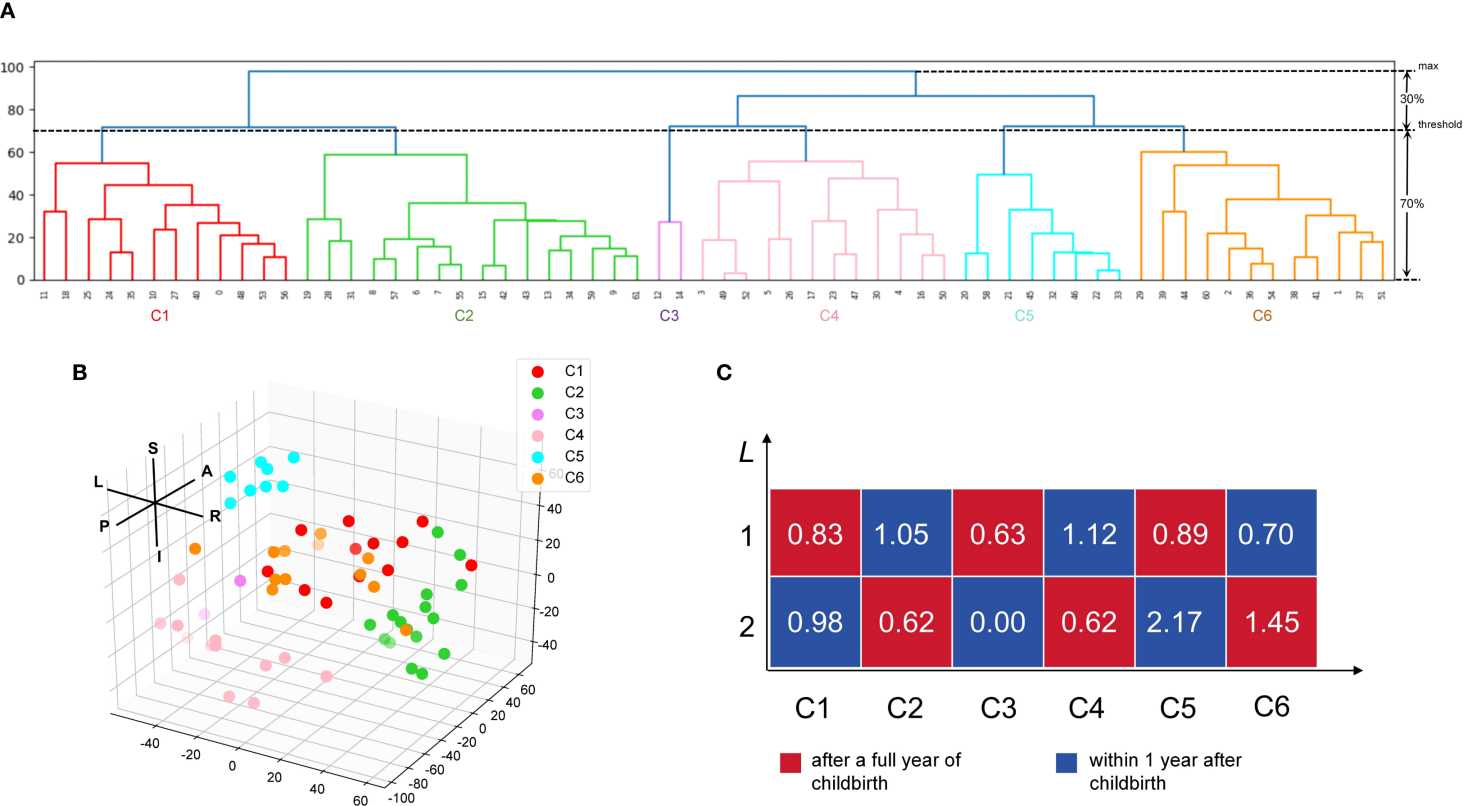
Hierarchy clustering analysis (HCA) of the extracted coordinate and the relationship between the postpartum period-triggered patterns and the clusters. **(A)** A dendrogram of the hierarchy clustering analysis (HCA). The HCA identified five clusters by setting the clustering threshold at 70% of the maximum distance between clusters. **(B)** Spatial location of the six clusters. Each dot indicates a specific coordinate that has been extracted. **(C)** Relatedness matrices for after a full year of childbirth and within 1 year after childbirth. The *L* score represents the relationship between the coordinate extracted from the postpartum period-triggered pattern and the cluster obtained from the HCA. L, left; R, right; A, anterior; P, posterior; S, superior; I, inferior; C, cluster.

**Figures 3A, B** depict the clustering patterns observed across the entire brain for the foci. According to the HCA results, the included coordinates were divided into 6 clusters (C1 to C6). C1 involved the left frontal lobe (medial and inferior frontal gyrus, Brodmann areas 10 and 46), extending to the caudate body and right frontal lobe. C2 was centered in the right frontal lobe (superior frontal gyrus, Brodmann area 10), precentral gyrus, and the posterior cingulate in the limbic system, as well as the hippocampus in the temporal lobe. C3 included the left temporal lobe (transverse temporal gyrus, Brodmann area 42), insula, thalamus, and superior temporal gyrus (Brodmann area 41). C4 was localized in the left temporal lobe (superior temporal gyrus) and occipital lobe (mid-occipital gyrus, cuneus), extending to the right occipital lobe. C5 involved the left frontal lobe (inferior frontal gyrus, Brodmann area 44), precentral gyrus, and the insula (Brodmann area 13). C6 was concentrated in the posterior cingulate in the limbic lobe, the right superior temporal gyrus, and the left occipital lingual gyrus. **Figure 3C** shows the correlation scores (*L*) of depression within 1 year postpartum and depression more than 1 year postpartum with these 6 clusters. Depression within 1 year postpartum exhibited higher *L* values than depression more than 1 year postpartum on C1, C2, C4 and C5 but lower *L* values than depression more than 1 year postpartum on C3 and C6.

## 4 Discussion

### 4.1 Enhanced functional activity in visual cortical areas in PPD

In our meta-analysis, increased functional activity was observed in the FFG.L and the MOG.L, areas crucial for emotion processing. The FFG.L is implicated in recognizing and expressing emotions (38), while the MOG.L is responsible for handling visual stimuli and spatial orientation (39). These findings suggest potential abnormalities in the neural circuits of the visual cortex in PPD patients. Consistent with previous studies, mothers who gave birth vaginally were more sensitive to infant cries compared to those who had cesarean sections. They also showed significantly increased activity in the FFG, which is associated with depressive states (40). Additionally, patients with somatic depression demonstrated enhanced FC between the INS.L and FFG.L, reflecting abnormal information transmission possibly related to altered emotional and cognitive experiences (41). The preferred information flow index in the MOG.L was decreased in patients with PPD, indicating a reduced ability to output information or an increased ability to receive information in this region (42). Depressed individuals who had experienced stressful life events displayed noticeably higher activation in the MOG.L compared to those who did not encounter such events (43). The study found that individuals at high risk for mood disorders showed increased activity in the FFG and MOG.L when discerning different moods (44). Avoidant individuals in negative parent-child relationships showed stronger activation features in the bilateral FFG and MOG (45). Another study has shown that connections between the higher-order visual cortex and AMYG play a crucial role in the identification and prioritization of negative emotional stimuli (46). This emphasizes the close relationship between the visual cortex and depressive emotions, suggesting that PPD patients may exhibit functional abnormalities in the visual cortex.

There are complex interactions between visual information processing and emotional regulation. Enhanced activity in the FFG and MOG may represent hyperactivity in interpreting social signals in PPD, potentially contributing to or exacerbating emotional problems. Besides, these findings may indicate hypersensitivity to negative visual stimuli in PPD, which is consistent with the well-recognized negative information processing biases in depression (47, 48). To deepen our understanding of how these brain regions relate to PPD, future research should explore how enhanced activity in visual processing regions specifically influences emotional and behavioral responses in PPD patients. What’s more, given that the FFG and MOG are also involved in maternal responses to infant faces and cries, our results may provide preliminary clues for future studies to explore whether altered visual cortical activity in PPD contributes to difficulties in mother-infant interaction (49, 50).

### 4.2 Decreased functional activity in PFC-the limbic system in PPD

Our results demonstrated the reduced function activity of ACG.L and STG.R in PPD. The PFC (especially ventral and medial regions) and the limbic system (including the cingulate gyrus) play key roles in processing emotional information, evaluating emotional responses, and regulating emotional expression (51, 52). This suggests potential abnormalities in the prefrontal cortical-limbic system functioning among PPD patients (53). The ACG.L is crucial for emotion modulation and cognitive control, being implicated in pain perception and processing memories associated with emotional experiences (54, 55). Reduced ACC activity has been observed in individuals with higher severity of internalizing symptoms during emotion regulation efforts (38). The ACG.L’s neurochemical activity is affected by hormonal factors, such as lower concentrations of glutamatergic complex metabolites associated with reduced HPA axis reactivity in PPD (56). Studies have indicated that progesterone can influence the neurochemical activity of the ACG (57). Pregnancy hormones, including estrogen and progesterone, play pivotal roles in triggering and regulating pregnancy-related neuroplasticity (58). Changes in the levels of estrogen and progesterone during the postpartum period have been proposed as potential factors contributing to postpartum mood swings and PPD (59).

The STG.R plays a role in emotion processing and emotion regulation. Studies have shown that its activity level is related to the degree of meditation, and among individuals with depression, its functional activity decreases in response to emotional stimuli. PPD has been linked to abnormal spontaneous neural activity in brain regions associated with mood and cognition. Specifically, mothers experiencing depression displayed noticeable increases in ReHo in the posterior cingulate and medial frontal regions. Simultaneously, there was a decrease in ReHo in the temporal gyrus (33).

### 4.3 Abnormal functional activity in motor sensory-related areas in PPD

In resting-state studies, the analysis revealed that compared to HCs, PPD patients showed decreased neurofunctional activity in the INS.R and the PreCG.R. Resting-state functional magnetic resonance imaging (rs-fMRI) can unveil brain regions associated with functional activity, particularly identifying differences in functional brain connectivity between patients and healthy individuals (60). Identifying neural network FC abnormalities and neural markers contribute to the identification of potential disease biomarkers, which are crucial for disease diagnosis, treatment, and prognosis (61).

The INS is a vital component of the emotional network, and is mainly responsible for emotion display and perception of unpleasant stimuli in the human brain, such as disgust (62). Studies have shown that the INS tends to have reduced activity in patients with recurrent major depression. Furthermore, variations in the VMHC of the INS lobe can serve as a potential neurobiological marker for major depression (63). Moreover, decreased ALFF has been observed in the bilateral INS and striatum in depressed individuals (64).

A meta-analysis conducted by Diener et al. emphasized the significant role of the anterior insular and rostral anterior cingulate cortex in depression, particularly in cognitive and emotional aspects (65). The PreCG consists of the primary motor cortex, and is responsible for voluntary movement. In addition to its motor functions, this region is intricately involved in cognitive processes and emotional regulation (66, 67). Previous studies have demonstrated that PPD patients exhibit lower dALFF variability in the sensorimotor network than HCs, particularly in the PreCG.R (30). Individuals at a higher risk of depression may show reduced activity in the PreCG.R during cognitive tasks (68). Moreover, depressed individuals with Alzheimer’s disease exhibit decreased ReHo in the PreCG.R (69), and reduced RSFC between the PreCG.R and the AMYG (70). Additionally, depressed patients demonstrate reduced gray matter volume in the PreCG.R compared to healthy individuals (71). Structural abnormalities in the PreCG.R among patients with PPD have been associated with impaired executive function (72). In summary, impairment in motor sensory-related regions may disrupt the integration of internal and external bodily signals, which could clinically manifest as slowed or poorly coordinated movements and reduced responsiveness to infant physical cues (73, 74). Such subtle sensorimotor deficits may interfere with daily functioning and mother-infant interactions(e.g., during feeding or holding) (75, 76), thereby presenting a novel dimension of the symptomatology of PPD that merits further investigation.

According to the HCA results, depression within 1 year postpartum showed higher *L* scores on C1, C2, C4 and C5 than depression more than 1 year postpartum. This may imply that functional abnormalities in bilateral frontal lobe, occipital lobe, and the limbic system regions may be associated with the onset and manifestation of depressive symptoms in patients with depression within 1 year postpartum. This suggested that the kind of patients may exhibit more dysfunction in individual emotion regulation, self-control, and attention control (77–79).

In contrast, depression more than 1 year postpartum showed higher *L* scores on C3 and C6 than depression within 1 year postpartum. This may imply that abnormalities in the functioning of the THA, INS, extending to the left temporal lobe region, may be more relevant to the onset and manifestation of depressive symptoms in patients with depression more than 1 year postpartum. The THA is an important relay station for afferent and efferent signals in the central part of the brain, and is involved in functions such as sensory transmission, emotion and pain regulation (80, 81). The temporal lobe is associated with functions such as social cognition and memory (82, 83). This suggested that depressed patients more than 1 year postpartum may be more likely to present with difficulties in interpersonal emotional processing, sensory transmission, and social cognitive dysfunction related (84).

Patients who were depressed within a year after giving birth had just experienced physical and psychological changes, such as hormonal fluctuations, sleep deprivation, pain, and fatigue (85). These factors may lead to impairments in self-regulation of emotions and self-control (86). Mothers at this stage may face mood swings, emotional outbursts, anxiety, irritability, etc., as it is difficult for them to cope with these changes and return to their pre-pregnancy physical and mental state (87). However, mothers who had been depressed for more than 1 year may had survived the initial physical and psychological changes and were adjusting to their new roles and responsibilities. A prolonged state of depression may lead to impaired sensory and interpersonal emotional processing, an inability to respond effectively to external emotional stimuli, and difficulties in expressing their feelings and needs. Depression within 1 year postpartum and depression more than 1 year postpartum showed different correlations in brain regions. This may reflect changes in the biological basis and neural mechanisms of PPD at different time points.

In conclusion, although the number of included studies was modest (10 studies; n=288), which may limit statistical power and generalizability, this limitation was mitigated by applying permutation-based statistical inference and jackknife sensitivity analyses within the AES-SDM framework. These robustness checks consistently supported the stability of our findings. Nevertheless, we acknowledge that the relatively small sample size remains a limitation, and future meta-analyses incorporating a larger number of datasets are warranted to further validate these results. In particular, future research should prioritize validation in larger independent cohorts to determine the reproducibility and generalizability of the identified alterations (e.g., in the MOG.L). Equally important, task-based fMRI paradigms targeting visual and emotion-regulation processes could directly probe the functional relevance of these regions, thereby clarifying their causal links to PPD symptomatology. Together, these directions would not only enhance the robustness of our findings but also accelerate their potential translation into clinically meaningful biomarkers.

## 5 Limitation

Although our meta-analysis explored alterations in brain function in PPD, several limitations warrant acknowledgment. Firstly, as an observational study, our analysis cannot establish causality directly. Heterogeneity in the design of included observational studies may have contributed to inconsistencies in results. Secondly, heterogeneity in study quality adds uncertainty to our findings. Despite our use of the NOS to assess study quality and efforts to control for confounders, uncontrolled variables may still influence results. While funnel plot analysis and Egger’s test showed no significant publication bias, we remain cautious about the potential impact of unpublished or incompletely reported studies on our results. Future studies should employ more robust designs and analyses, especially those probing causality directly. Improved control of confounding factors and systematic exploration of unpublished studies are crucial for enhancing research reliability and depth. Due to the small sample size of included studies, the conclusion of the study should be treated with caution. In subsequent research, we plan to use the meta-analysis results as a foundation and apply them to specific clinical studies to validate these findings’ effectiveness and reliability.

## Supplementary material

Supplementary Figure 1 Funnel plots of the increased and decreased effect-sizes in the meta-analysis.

Supplementary Table 1 PRISMA 2020 checklist.

Supplementary Table 2 PubMed search process.
