# Supplementary material for: Postpartum depression-associated localized neural dysfunction: a voxel-wise meta-analysis of amplitude and synchronization alterations in resting-state fMRI
Source: Front Psychiatry. 2025 Oct 8;16:1660550. doi: 10.3389/fpsyt.2025.1660550 (PMC12540480; doi:10.3389/fpsyt.2025.1660550)

Supplementary Figures

Supplementary Figure 1: Funnel plots of the increased and decreased effect-sizes in the meta-analysis (A: Left fusiform gyrus, Eggers' test p-value = 0.872; B: Left middle occipital gyrus, Eggers' test p-value = 0.810; C: Corpus callosum, Eggers' test p-value = 0.553; D: Left cerebellum, hemispheric lobule VIII, Eggers' test p-value = 0.340; E: Left anterior cingulate/paracingulate gyri, Eggers' test p-value = 0.644; F: Right superior temporal gyrus, Eggers' test p-value = 0.560; G: Right insula, Eggers' test p-value = 0.497; H: Right precentral gyrus, BA 6, Eggers' test p-value = 0.096).

The horizontal axis represents the effect-size. The vertical axis represents the standard.

A

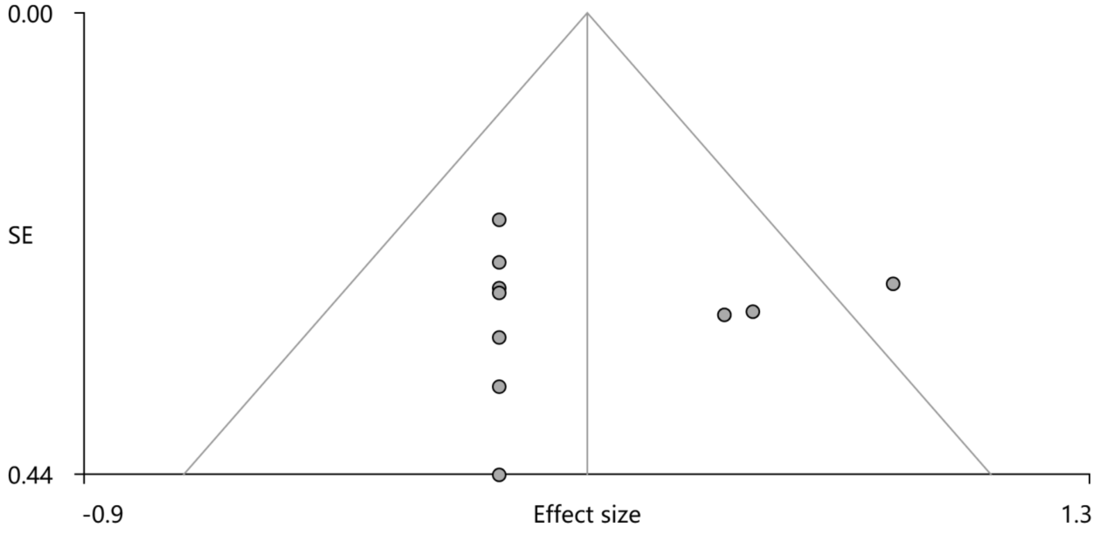

B

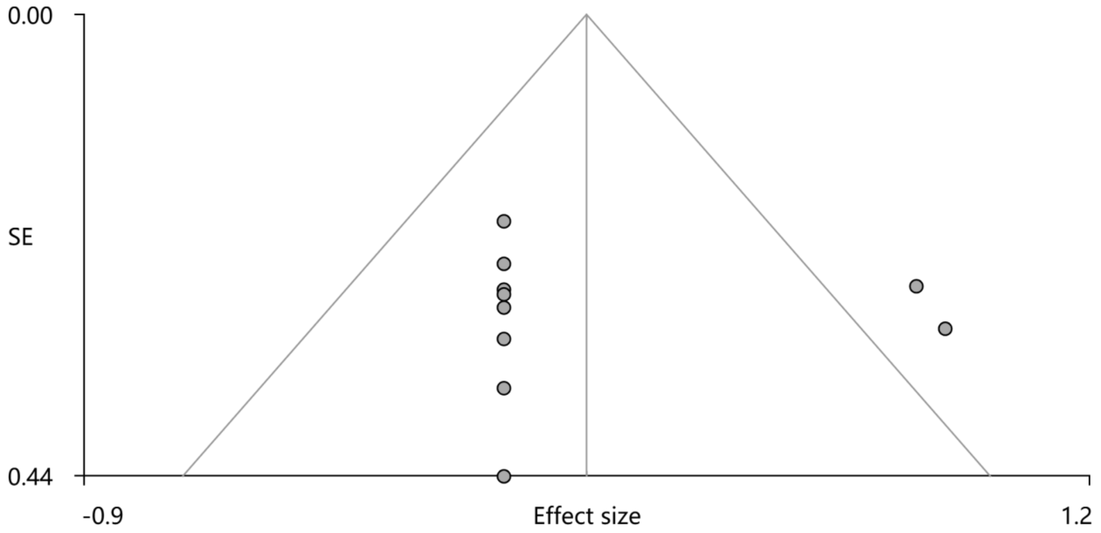

C

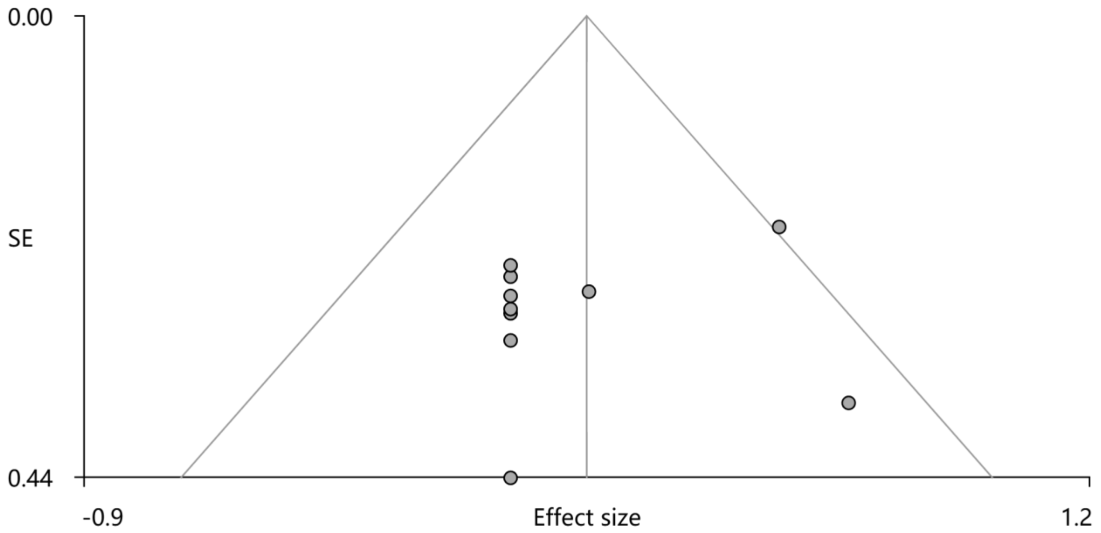

D

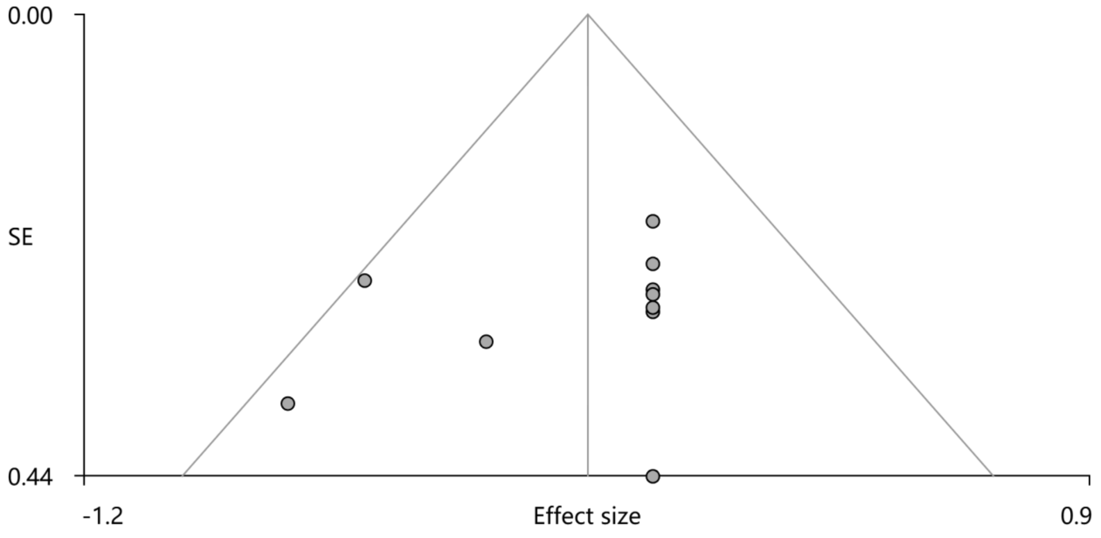

E

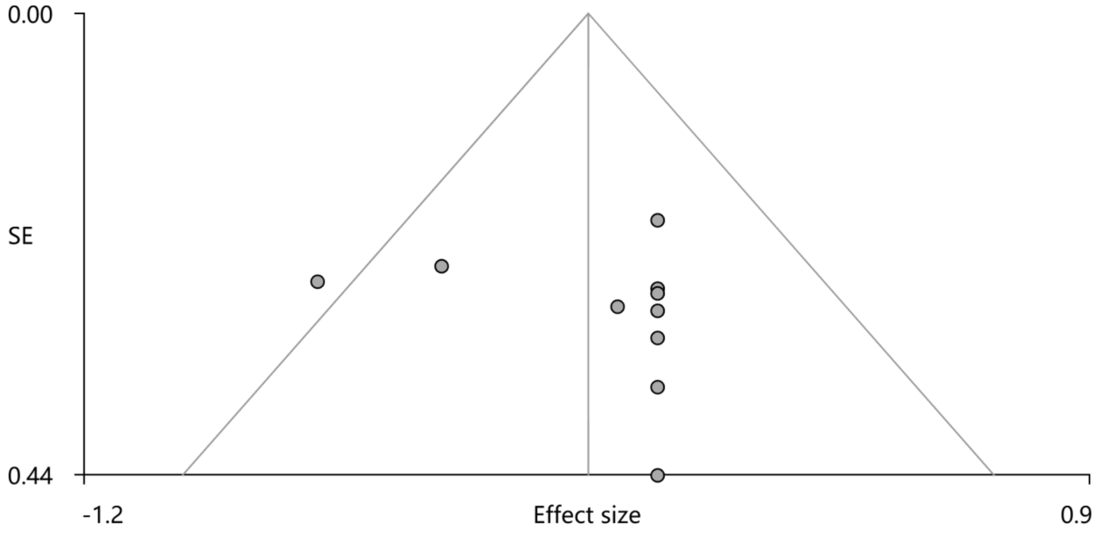

F

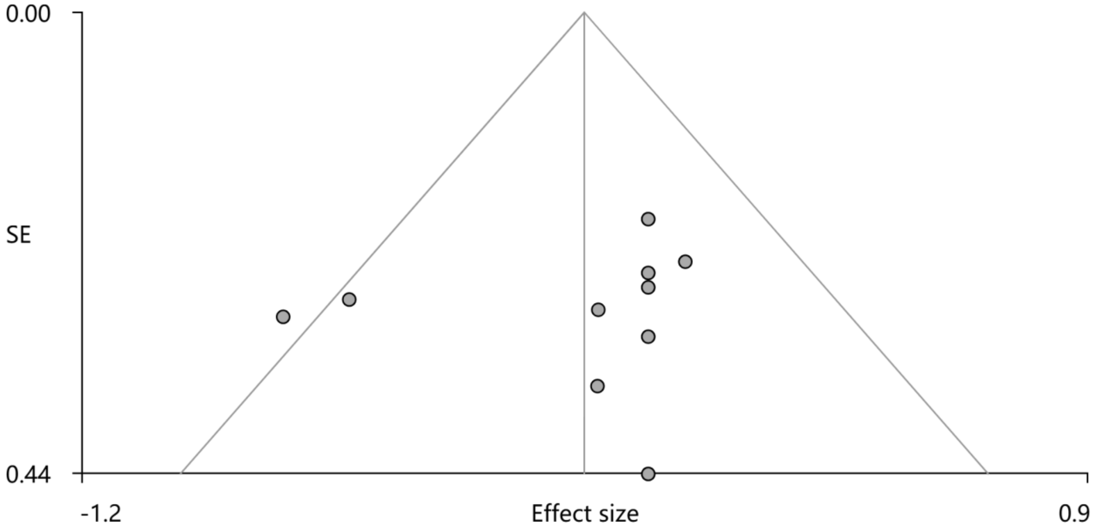

G

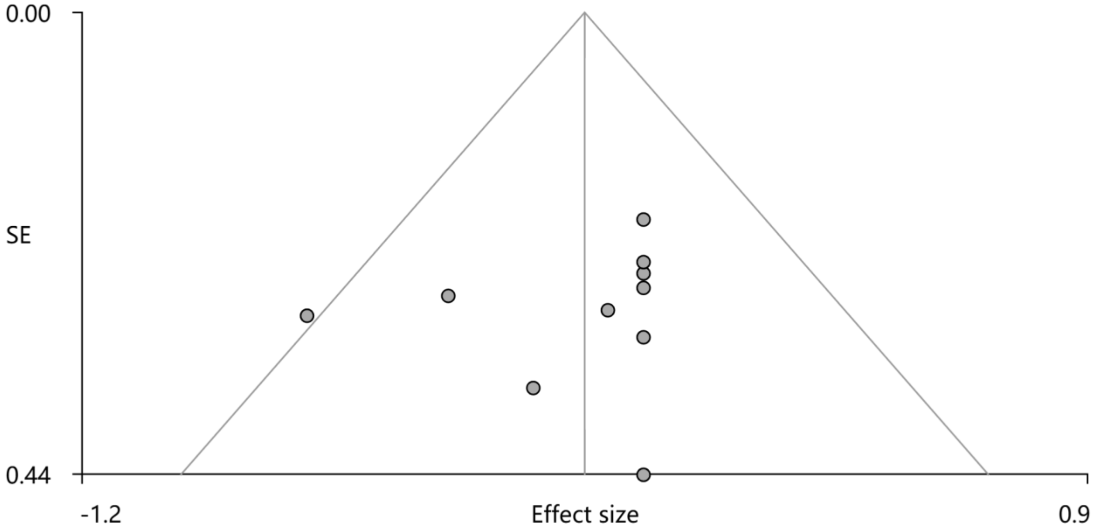

H

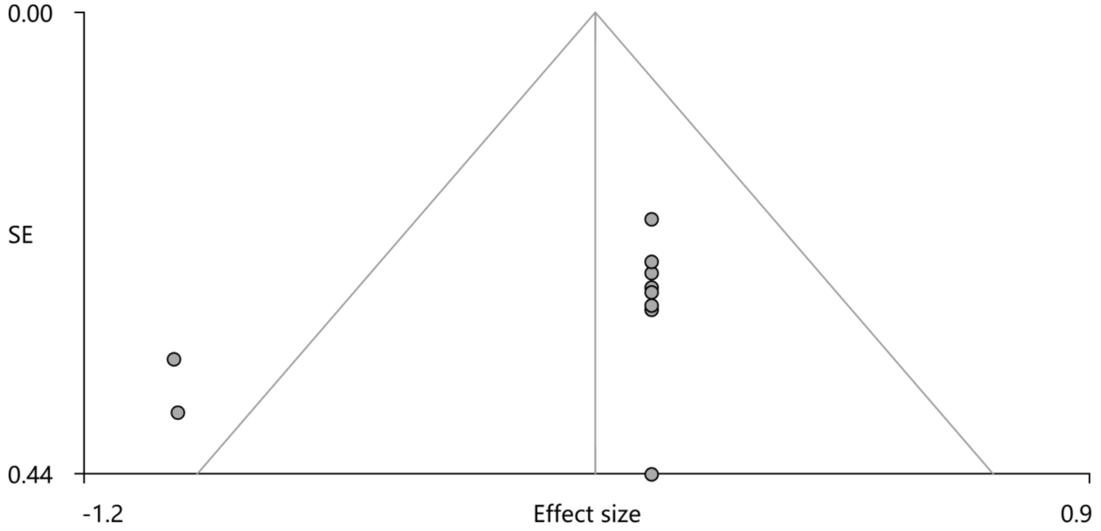

Supplement: Supplementary Figure 1 — Funnel plots of the increased and decreased effect-sizes in the meta-analysis. [file Image1.pdf]
