# Supplementary material for: Postpartum depression-associated localized neural dysfunction: a voxel-wise meta-analysis of amplitude and synchronization alterations in resting-state fMRI
Source: Front Psychiatry. 2025 Oct 8;16:1660550. doi: 10.3389/fpsyt.2025.1660550 (PMC12540480; doi:10.3389/fpsyt.2025.1660550)
Supplement: Supplementary Table 2 — PubMed search process. [file Table2.docx]

**Supplementary Table 2: PubMed search process**

| Serial number | Search items |  |
| --- | --- | --- |
| #1 | "depression, postpartum"[MeSH Terms] | 7751 |
| #2 | "postnatal depression"[Title/Abstract] OR "depression postnatal"[Title/Abstract] OR "post partum depression"[Title/Abstract] OR "depression post partum"[Title/Abstract] OR "post partum depression"[Title/Abstract] OR "postpartum depression"[Title/Abstract] OR "post natal depression"[Title/Abstract] OR (("depressed"[All Fields] OR "Depression"[MeSH Terms] OR "Depression"[All Fields] OR "depressions"[All Fields] OR "depression s"[All Fields] OR "depressive disorder"[MeSH Terms] OR ("depressive"[All Fields] AND "disorder"[All Fields]) OR "depressive disorder"[All Fields] OR "depressivity"[All Fields] OR "depressive"[All Fields] OR "depressively"[All Fields] OR "depressiveness"[All Fields] OR "depressives"[All Fields]) AND "Post-Natal"[Title/Abstract]) OR "post natal depression"[Title/Abstract] | 9809 |
| #3 | #1OR #2 | 12056 |
| #4 | "Magnetic Resonance Imaging"[MeSH Terms] | 544722 |
| #5 | "imaging magnetic resonance"[Title/Abstract] OR "nmr imaging"[Title/Abstract] OR "imaging nmr"[Title/Abstract] OR "tomography nmr"[Title/Abstract] OR "tomography mr"[Title/Abstract] OR "mr tomography"[Title/Abstract] OR "nmr tomography"[Title/Abstract] OR ("magnetic resonance imaging"[MeSH Terms] OR ("Magnetic"[All Fields] AND "Resonance"[All Fields] AND "Imaging"[All Fields]) OR "magnetic resonance imaging"[All Fields] OR ("Steady"[All Fields] AND "State"[All Fields] AND "Free"[All Fields] AND "Precession"[All Fields] AND "MRI"[All Fields]) OR "steady state free precession mri"[All Fields]) OR "steady state free precession mri"[Title/Abstract] OR "Zeugmatography"[Title/Abstract] OR "imaging chemical shift"[Title/Abstract] OR "chemical shift imagings"[Title/Abstract] OR (("Image"[All Fields] OR "image s"[All Fields] OR "imaged"[All Fields] OR "imager"[All Fields] OR "imager s"[All Fields] OR "imagers"[All Fields] OR "Images"[All Fields] OR "Imaging"[All Fields] OR "imaging s"[All Fields] OR "Imagings"[All Fields]) AND "chemical shift"[Title/Abstract]) OR "shift imaging chemical"[Title/Abstract] OR ((("Shift"[All Fields] OR "shifted"[All Fields] OR "shifting"[All Fields] OR "shiftings"[All Fields] OR "shifts"[All Fields]) AND ("Image"[All Fields] OR "image s"[All Fields] OR "imaged"[All Fields] OR "imager"[All Fields] OR "imager s"[All Fields] OR "imagers"[All Fields] OR "Images"[All Fields] OR "Imaging"[All Fields] OR "imaging s"[All Fields] OR "Imagings"[All Fields])) AND "Chemical"[Title/Abstract]) OR "chemical shift imaging"[Title/Abstract] OR "magnetic resonance image"[Title/Abstract] OR "image magnetic resonance"[Title/Abstract] OR "magnetic resonance images"[Title/Abstract] OR "resonance image magnetic"[Title/Abstract] OR "magnetization transfer contrast imaging"[Title/Abstract] OR "mri scans"[Title/Abstract] OR "mri scan"[Title/Abstract] OR "scan mri"[Title/Abstract] OR "scans mri"[Title/Abstract] OR (("tomographie"[All Fields] OR "Tomography"[MeSH Terms] OR "Tomography"[All Fields] OR "tomographies"[All Fields] OR "tomography s"[All Fields] OR "tomography, x ray computed"[MeSH Terms] OR ("Tomography"[All Fields] AND "x ray"[All Fields] AND "computed"[All Fields]) OR "x-ray computed tomography"[All Fields] OR "tomographys"[All Fields]) AND "proton spin"[Title/Abstract]) OR "proton spin tomography"[Title/Abstract] OR "fMRI"[Title/Abstract] OR "mri functional"[Title/Abstract] OR "functional mri"[Title/Abstract] OR "functional mris"[Title/Abstract] OR ("MRIs"[All Fields] AND "Functional"[Title/Abstract]) OR "functional magnetic resonance imaging"[Title/Abstract] OR "magnetic resonance imaging functional"[Title/Abstract] OR "spin echo imaging"[Title/Abstract] OR "echo imaging spin"[Title/Abstract] OR ((("Echo"[Journal] OR "Echo"[All Fields]) AND ("Image"[All Fields] OR "image s"[All Fields] OR "imaged"[All Fields] OR "imager"[All Fields] OR "imager s"[All Fields] OR "imagers"[All Fields] OR "Images"[All Fields] OR "Imaging"[All Fields] OR "imaging s"[All Fields] OR "Imagings"[All Fields])) AND "Spin"[Title/Abstract]) OR "imaging spin echo"[Title/Abstract] | 708211 |
| #6 | #4OR#5 | 708211 |
| #7 | #3AND#6 | 108 |
| #8 | "fMRI" OR" Functional magnetic resonance imaging* "OR "functional MRI" OR" BOLD "OR "Blood oxygen level dependent "OR "regional homogeneity "OR "ReHo "OR "amplitude of low-frequency fluctuation* "OR "ALFF "OR "fractional amplitude of low-frequency fluctuation* "OR "fALFF "OR "brain activity "OR "brain function* "OR "VMHC "OR "voxel-mirrored homotopic connectivity "OR "resting state* "OR "rs " | 266445 |
| #9 | #7AND#8 | 62 |
