# Supplementary material for: Postpartum depression-associated localized neural dysfunction: a voxel-wise meta-analysis of amplitude and synchronization alterations in resting-state fMRI
Source: Front Psychiatry. 2025 Oct 8;16:1660550. doi: 10.3389/fpsyt.2025.1660550 (PMC12540480; doi:10.3389/fpsyt.2025.1660550)
Supplement: Supplementary file 4 [file Table3.docx]

Supplementary Table 3: Criteria for objective assessment of methodological quality of individual studies.

| **Category 1: Sample characteristics (10)** |
| --- |
| 1. Patients were evaluated with specific standardized diagnostic criteria (1) |
| 1. Important demographic data (age and gender) were reported with mean (or median) and standard deviations (or range)) (2) |
| 1. Healthy comparison subjects were evaluated to exclude psychiatric and medical illnesses and demographic data was reported (1) |
| 1. Important clinical variables (e.g. illness duration, medication status, HAMA scores, HAMD scores) were reported with mean (or median) and standard deviations (or range)) (4) |
| 1. Sample size per group > 10 (2) |
| **Category 2: Methodology and reporting (10)** |
| 1. Whole brain analysis was automated with no a-priori regional selection (3) |
| 1. Magnet strength at least 1.5T (1) |
| 1. At least 5 minutes of resting state acquisition (1) |
| 1. Whole brain coverage of resting scans (1) |
| 1. The acquisition and preprocessing techniques were clearly described so that they could be reproduced (1) |
| 1. Coordinates reported in a standard space (1) |
| 1. Significant results are reported after correction for multiple testing using a standard statistical procedure (FDR, FWE or permutation-based methods) (1) |
| 1. Conclusions were consistent with the results obtained and the limitations were discussed (1) |

A maximum score of 20 for each study, allocated as per the criteria specified above. Each of the two sections, sample characteristics, methodology and reporting, is worth 10 points, and the value following each item represents the score that the item would have received if it had met the requirements. Take the first project as an example, patients were assessed in a study using specific standardized diagnostic criteria, then the study would receive a score of 1.
